# A practical scoring system to predict mortality in patients with perforated peptic ulcer

**DOI:** 10.1186/s13017-015-0008-7

**Published:** 2015-02-21

**Authors:** Ebru Menekse, Belma Kocer, Ramazan Topcu, Aydemir Olmez, Mesut Tez, Cuneyt Kayaalp

**Affiliations:** Department of General Surgery, Ankara Numune Training and Research Hospital, Ankara, 06100 Turkey; Department of General Surgery, Faculty of Medicine, Sakarya University, Sakarya, 54000 Turkey; General Surgery Clinic, Turhal State Hospital, 60300 Tokat, Turkey; Department of Surgery, Faculty of Medicine, Mersin University, 33343 Mersin, Turkey; Department of Surgery, Faculty of Medicine, Inonu University, 44280 Malatya, Turkey

**Keywords:** Peptic ulcer, Perforation, Mortality, Scoring methods

## Abstract

**Introduction:**

The mortality rate of perforated peptic ulcer is still high particularly for aged patients and all the existing scoring systems to predict mortality are complicated or based on history taking which is not always reliable for elderly patients. This study’s aim was to develop an easy and applicable scoring system to predict mortality based on hospital admission data.

**Methods:**

Total 227 patients operated for perforated peptic ulcer in two centers were included. All data that may be potential predictors with respect to hospital mortality were retrospectively analyzed.

**Results:**

The mortality and morbidity rates were 10.1% and 24.2%, respectively. Multivariated analysis pointed out three parameters corresponding 1 point for each which were age >65 years, albumin ≤1,5 g/dl and BUN >45 mg/dl. Its prediction rate was high with 0,931 (95% CI, 0,890 to 0,961) value of AUC. The hospital mortality rates for none, one, two and three positive results were zero, 7.1%, 34.4% and 88.9%, respectively.

**Conclusion:**

Because the new system consists only age and routinely measured two simple laboratory tests (albumin and BUN), its application is easy and prediction power is satisfactory. Verification of this new scoring system is required by large scale multicenter studies.

## Introduction

In treatment of peptic ulcer, incidence of elective surgery tended to decrease due to eradication of *Helicobacter pylori* during the recent three decades whereas incidence of emergency surgical interventions for complications of the disease did not decrease [1-3]. Moreover, population ageing and extensive use of non-steroid anti-inflammatory drugs increased the incidence of bleeding and perforation of peptic ulcer [1]. Only 5-10% of the patients with bleeding peptic ulcers require surgical intervention whereas almost all patients with perforated peptic ulcer (PPU) necessitate surgery [1]. The risk of mortality (6-30%) and morbidity (21-43%) at PPU unfortunately have not changed during the last decades [1,3-6]. Perforation was the cause of death in 70% of the patients with peptic ulcer and rate of mortality due to PPU is 10-fold higher than other acute abdominal factors such as acute appendicitis and acute cholecystitis [7].

Some scoring systems such as Boey, Peptic Ulcer Perforation Score (PULP) and ASA (American Society of Anesthesiologists) have been already developed for prediction of mortality at PPU [5,8,9]. PULP score appears to have the greatest predictability of mortality however it is impractical with its complexity [5]. Boey score is a more practical but its predictability value was found varying in several studies [5,10-12]. Both scoring systems require a well history taking to detect the duration of symptoms and co-morbidities [5,8]. However, those data cannot be taken reliably from some elderly patients. ASA as a scoring system is non-specific for PPU, its predictability is not superior than the others and its major drawback is its subjective assessment [5,10]. Detection of patients with high risk for mortality after PPU surgery can allow other treatment modalities except surgery or can necessitate some extra care protocols to decrease the mortality [6].

Our aim was to develop a new and easy applicable scoring system to predict mortality at PPU patients.

### Patients and methods

The records of surgically treated PPU patients at Ankara Numune Training and Research Hospital and Inonu University Faculty of Medicine between dates 2009 and 2010 were reviewed as retrospectively. The computerized and documentary archives of patients in both of hospital were used in this study. The cases with malignant perforated tumors, marginal ulcer or incomplete data were excluded from the analysis.

The patients were diagnosed according to preoperative clinical features, routine laboratory tests, radiological findings and operative evidence. All the procedures were conducted via an open surgical approach.

The following data were collected: age, gender, white blood cell count (WBC), hemoglobin (Hb), urea, creatinine (Cre), albumin (Alb), systolic blood pressure (BP-S), diastolic blood pressure (BP-D), mean arterial pressure (MAP), pulse, perforation size, admission duration, ASA, Boey, PULP scores, duration of operation, medical illnesses, postoperative complications, reasons of mortality. Laboratory data’s were used at the time of admission. The death that occurred within 30 days after surgical treatment or death at the same admission was defined as hospital mortality. The time interval longer than 24 hours between presumed perforation and surgery was accepted as a delayed admission. Factors associated with mortality and morbidity were analyzed using univariate and multivariate analysis. A clinical POMPP (Practical scoring system of mortality in patients with perforated peptic ulcer) score based on the final logistic regression model was constructed for mortality. Additionally, logistic regression analysis and receiver-operating characteristic (ROC) curve analysis were used to calculate risk predictions for mortality in Boey, PULP and ASA scoring systems and their predictability on mortality was compared with the new scoring system. The definitions of the mentioned scoring systems are presented in the Table 1.

**Table 1:** **Comparison of scoring systems contents for mortality in patients with peptic ulcer perforation**

**Scoring systems**	**PULP** - **points**		**ASA** - **scores**		**BOEY** - **points**		**POMPP** - **points**	
**Substances**	Age > 65	3	Normal health	1	Medical illness	1	Age > 65	1
	Comorbid active malign disease or AIDS	1	Mild systemic disease	2	Preoperative shock	1	BUN > 45 mg/dl	1
	Comorbid liver cirrhosis	2	Severe systemic disease	3	Duration of peptic ulcer perforation > 24 h	1	Albumin < 1.5 g/L	1
	Concomitant use of steroids	1	Severe systemic disease with a constant treat to life	4				
	Shock	1	Not expected survival for patients without surgery	5				
	Perforation time on admission >24	1						
	Serum creatinine >1.47 mg/dl	2						
	ASA 2	1						
	ASA 3	3						
	ASA 4	5						
	ASA 5	7						
**High score**	>6		>3		>1		>1	
**Total score**	0-18		1-5		0-3		0-3	

Perforation longer than 24 hours was differently defined by PULP and Boey scorings. This term was defined as time interval from perforation (onset of symptoms or aggravation of symptoms) until admission to hospital for PULP [5] whereas this term was defined as the time interval from perforation until surgery for Boey [13]. We also used the definition of Boey scoring system for perforation duration in all scorings. Therefore, total score of PULP may have resulted higher in our study than the original application.

Preoperative shock was defined as blood pressure < 100 mm Hg and heart rate >100 beats/ min for PULP whereas this term was described as only blood pressure < 100 mm Hg for Boey [5,13]. The parameter of preoperative shock was defined compatible with original form of each study in evaluation of these scoring systems in our study.

### Statistical analysis

Shapiro-Wilk test was used for assessing normality. Continuous data are presented as mean ± SD while differences between groups were analyzed by means of Students *t* test. Categorical variables were analyzed with χ2 tests. Logistic regression was used to identify variables associated with mortality. Variables with p ≤ 0.2 in the univariate analyses were included in multivariate analyses. Results of the multivariable analysis were shown as odds ratio (OR) and corresponding 95% confidence interval (CI). The analysis of the ROC curve used to define the optimal cut-off value for continuous variables in mortality. A clinical score based on the final logistic regression model was constructed; 1 point was given to indicate presence of each predictive factor.

Model discrimination was measured by the area under the receiver–operator characteristic (ROC) curve (AUC). The discrimination of a prognostic model is considered perfect, good, moderate and poor for AUC values of 1; >0,8; 0,6–0,8 and <0,6; respectively.

## Results

We enrolled 325 patients underwent surgical treatment for PPU. A total of 98 patients were excluded because the fulfilled at least one of the exclusion criteria. The study population included remaining 227 patients with a mean age of 50.6 ± 19.6 (ranged16-95) years. Table 2 shows the clinical characteristics of PPU patients and comparison of these characteristics for mortality and morbidity according to univariate analysis. Hospital mortality was 10.1% (n: 23) in the patients while pneumonia, myocardial failure combined with arrhythmia, septicemia and renal failure were found in 15, five, two and one patients, respectively. Morbidity rate was 24.2% (n: 55). The morbidities were pulmonary failure (n:24), wound infection (n:23), evisceration (n:10), renal failure (n:7), postoperative ileus (n:6), cardiac failure (n:5), suture leakage (n:3) and intraabdominal abscess (n:2)., respectively. Mean length of hospital stay was 7.9 ± 9.0 days (ranged 1–115).

**Table 2:** **Clinical characteristics of patients in terms of mortality**

**Variable**	**Mortality**	**No Mortality**	***P***	**Morbidity**	**No morbidity**	***P***
	**n** **=** **23**	**n** **=** **204**		**n** **=** **55**	**n** **=** **172**	
Age (years) (mean ± SD)	74.5 ± 12.1	47.9 ± 18.4	<0.0001	61.4 ± 18.1	47.2 ± 18.8	<0.0001
Sex; Male/Female (n,%)	18(9.1)/5(15.6)	180(90.9)/27(84.4)	NS^†^	43(21.7)/12(37.5)	155(78.3)/20(62.5)	0.047
White blood cell count (10/μL) (mean ± SD)	12.5 ± 7.8	13.7 ± 6.5	NS^†^	146.9 ± 86.7	132.0 ± 59.8	NS^†^
Hemoglobin (g/dl) (mean ± SD)	12.8 ± 2.8	15 ± 2.3	<0.0001	14.3 ± 3.2	14.9 ± 2.1	NS^†^
BUN (mg/dl) (mean ± SD)	123.5 ± 85.9	36.6 ± 20.9	<0.0001	70.5 ± 67.1	37.4 ± 26.2	<0.0001
Creatinine (mg/dl) (mean ± SD)	2.71 ± 2.07	1.15 ± 0.86	<0.0001	1.78 ± 1.32	1.20 ± 1.21	0.003
Albumin (g/L) (mean ± SD)	1.52 ± 0.51	2.57 ± 0.75	<0.0001	2.45 ± 0.69	3.12 ± 0.73	<0.0001
BP-S*(mm/Hg) (mean ± SD)	107 ± 28.4	125.9 ± 21.7	<0.0001	124.1 ± 28.6	123.9 ± 21.2	NS†
BP-D**(mm/Hg) (mean ± SD)	67.2 ± 19.4	76.7 ± 13.6	0.003	77.2 ± 16.9	75.3 ± 13.8	NS†
MAP***(mmHg) (mean ± SD)	80.4 ± 21.8	93.1 ± 15.03	<0.0001	92.8 ± 19.7	91.5 ± 14.9	NS†
Pulse (/ min) (mean ± SD)	113.2 ± 30.2	94.7 ± 14.3	<0.0001	104.6 ± 22.3	93.8 ± 14.7	<0.0001
Time from perforation to surgery (h) (n, %)						
<24 h	1 (1.2)	80 (98.8)	0.001	28 (17.4)	133 (82.6)	<0.0001
>24 h	22 (15.1)	124 (84.9)		27 (39.7)	41 (60.3)	
Perforation size (cm) (n, %)						
<0.5	15 (8.9)	153 (91.1)	0.02	4 (13.8)	25 (86.2)	0.001
0.5-1	1 (3.4)	28 (96.6)		36 (21.1)	135 (78.9)	
>1	7 (23.3)	23 (76.7)		15 (50)	15 (50)	
Operation time (min) (mean ± SD)	103.3 ± 42.4	81.6 ± 28.1	0.001	95.7 ± 46.8	80.3 ± 22.8	0.002
Other medical illnesses (n,%)						
Absent	4 (2.5)	155 (97.5)	<0.0001	22 (13.6)	140 (86.4)	<0.0001
Present	19 (27.5)	49 (72.1)		33 (48.5)	35 (51.5)	

*BP-*S*: Blood pressure-systolic, **BP-*D*: Blood pressure-diastolic, ***MAP: Median artery pressure, †*NS*: Non-significant.

The operative procedures included mainly simple closure (n: 218) or some definitive procedures (n:9) such as pyloroplasty or gastrectomy in cases of accompanying hemorrhage, large or multiple perforations.

Three variables were statistically significant in multivariate analysis: albumin level equal or less than 1.5 (OR = 0.0445), age over 65 (OR =1.1258), and BUN level higher than 45 (OR = 1.0353) (Table 3). A probability score was calculated by adding points given to these variables. Despite the differences in regression coefficients, 1 point was given for each of these risk factors to simplify procedure. The resulting predicting of mortality in perforated peptic ulcer (POMPP) score ranged between score 0 to 3.

**Table 3:** **Independent predictor of mortality identified by multivariate logistic regression analysis**

**Predictors**	***P*** **value**	SE*	**Odds ratio**	**95** **%** **Cl**
Albumin	0.0005	0.89	0.0445	0.0077 to 0.2577
BUN	0.0003	0.009	1.0353	1.0160 to 1.0550
Age	0.0013	0.03	1.1258	1.0474 – 1.2100

*Standard Error.

Three groups of patients were defined based on the POMPP score. In the first group, with a score 0, there was no mortality. The second group included patients with POMPP score 1, who had a 7.1% risk of mortality; this group comprised approximately 1.8% of the cohort. The third group, comprising approximately 4.8% of the patients, included those with a POMPP score of 2 whose risk of mortality 34.4% and last group with a POMPP score of 3 who had an 88.9% risk of mortality, this group comprised about 3.5% of the cohort (Table 4).

**Table 4:** **Risk of mortality according to the POMPP score in patients with peptic ulcer perforation**

**POMPP Score**	**No Mortality**	**Mortality**
	**n** **(%)**	**n** **(%)**
**0**	130 (100)	0 (0)
**1**	52 (92.9)	4 (7.1)
**2**	21 (65.6)	11 (34.4)
**3**	1 (11.1)	8 (88.9)

**Figure 1:**
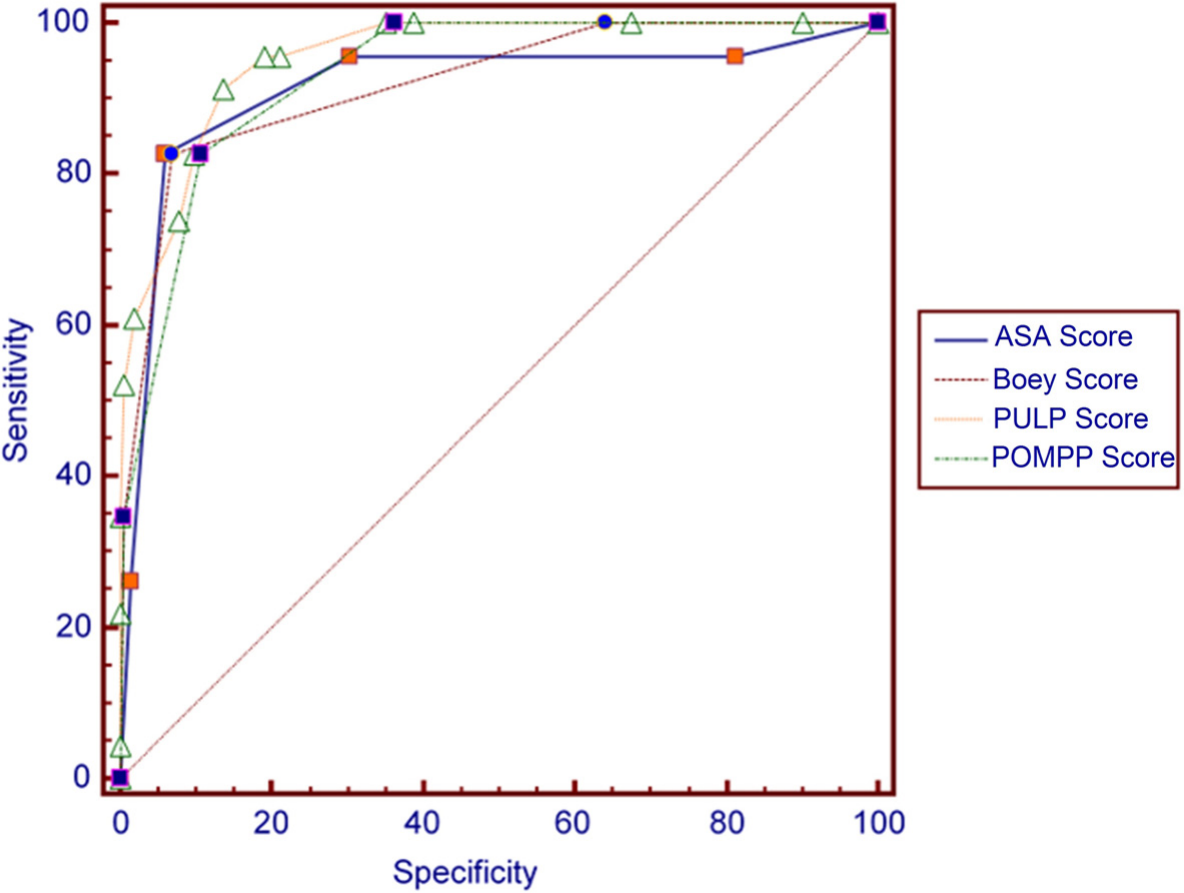
**ROC curves analysis of POMPP** **,** **PULP** **,** **Boey and ASA scoring system** **.**

The area under the ROC curve (AUC) was 0.931 (95% CI, 0.89-0.96) (Figure 1). The AUC values of the other scoring systems evaluated in our study have been presented in Table 5. The specificity, sensitivity, negative likelihood and positive likelihood ratios for POMPP score exceeding 1 point were 89.2%; 82.6%; 0.19 and 7.66, respectively.

**Table 5:** **The ROC curves results of different scoring system for mortality in peptic ulcer perforation**

**Scoring Systems**	**AUC**	SE*	**95** **%** **Cl**
**ASA**	0.914	0.0401	0.870 to 0.947
**BOEY**	0.920	0.0282	0.876 to 0.952
**PULP**	0.955	0.0164	0.919 to 0.978
**POMPP**	0.931	0.0195	0.890 to 0.961

*Standard Error.

## Discussion

We described a new and easily applicable scoring system to predict the postoperative mortality rate in patients with PPU. This scoring system simply based on only age and routinely measured two simple laboratory tests (albumin, BUN). Similarly to us, PULP or Boey scores were found that age over 65 or 60 was an independent risk factor for mortality [5,13]. Advanced age had been reported in several studies as an independent risk factor on mortality in PPU patients [4,14-18] and its importance is still remains [16,19].

Another parameter of POMPP scoring system was BUN level which is regulated as a result of several conditions such as protein catabolism, steroid intake and gastrointestinal bleeding. Regardless of renal functions, it is also accepted as a marker of a severity of disease [20]. In the study of Khuri et al., BUN > 40 mg/dl was found as a risk factor that increases 30-day mortality after non-cardiac operations [21]. In PULP and Jabolpur scoring systems, high level of serum creatinine was used in predicting risk for mortality [5,12]. In the study of Thorsen et al., serum creatinine level over 1.33 mg/dl was detected as an independent risk factor that indicates mortality risk in PPU [11]. Additionally, it was stated in this study that hypoalbuminemia and high creatinine levels may reflect some underlying pathologies and diseases such as presence of cancer, chronic severe disease and acute diseases that may cause dehydration or accompany with infection and sepsis [11]. We considered that high predictive power of low albumin and high BUN levels as well as advanced age in mortality is associated with the broad spectrum of underlying pathological events and diseases. Hypoalbuminemia alone had been shown as marker of increased risk of morbidity and mortality in PPU patients [22]. Thorsen et al. was found that hypoalbuminemia was a strong factor which might determine mortality solely (AUC: 0.78) [11]. Strong correlation between hypoalbuminemia and mortality in PPU patients is not surprising when reduction of albumin synthesis is considered in cases of dehydration, hepatic dysfunction, cancer, critical clinical course, systemic inflammatory response syndrome and sepsis [22,23].

PULP scoring system was constructed by testing a large patient population as the national data [5]. Even though, mortality predictive power of PULP scoring system was a little better than ours (PULP AUC: 0,955 vs. POMPP AUC: 9,931; p > 0,5), it is not easy to use the PULP in clinical practice. PULP is based on partially anamnesis and admission time was defined as the end of time interval which didn’t reflect total duration of abdominal contamination. Additionally, three variables including missing data more than 20% were excluded from the PULP study and some more missing data below 20% were included in. Moller et al. was given AUC value of 0.83 for mortality prediction for PULP scoring system [5]. In a recent study by Thorsen et al. found the AUC value as 0.79 [11], whereas we have found it as 0.95. While calculating the PULP score in our study, we modified the defined time interval as from perforation onset to the surgery. For this reason, prediction of PULP in our analysis might be higher than the previously reported ones.

The other defined scoring system of Boey is more practical than the PULP. However, prediction values of Boey scoring system were quite varying in several studies as AUC values ranged between 0, 63 to 0, 86 [5,11,12,15,24]. In our analysis we found a better Boey value (AUC: 0.92) for prediction then the reported ones. On the other hand, Boey scoring system didn’t involve advanced age which is generally an important parameter for mortality in PPU [8]. Exclusion of advanced age might be caused by the fact that this scoring system was defined three decades before. Incidence of PPU complications increased in the population of advanced age due to prolonging mean lifetime in the present time and increased use of NSAIs in the advanced ages [25].

Several studies were analyzed the mortality prediction of ASA status in PPU patients and found AUC values between 0, 73 and 0, 91 [5,9,11,15]. ASA is not specific scoring system for neither PPU and it is mainly based on the co-morbid diseases and their severity [11,25,26]. Although co-morbidities are important risk factors for mortality, under diagnosed or unknown chronic diseases on emergency admission can result to underscoring of ASA. On the other hand, sepsis is as important as the additional medical diseases on the mortality of PPU [4,6,9]. Beside all that, the main problem of ASA scoring is that calculation is performed subjectively and differences between interpretations may be observed [10].

In fact, all of the scoring system models compared in our study had similar and well predictive power for mortality in PPU patients. None of the previously described scoring systems were widely accepted in clinical practice yet. The reason can be their complexity, non-specificity or confused and subjective points in the mind of clinicians such as definitions of preoperative shock, perforation duration and severity of medical illnesses. We believe that three very clear parameters (age, albumin and BUN) can be easily adopted in the clinical practice to predict the surgical mortality of PPU patients. Respiratory support, circulatory stabilization, preoperative and postoperative care in ICU, frequent monitorization and perioperative care protocols can be added to the high risk patients with PPU [5,6]. It is demonstrated that if the high risk patients got extra perioperative care, the hospital mortality rate could be reduced from the standard care patients (17% and 27%, respectively, *p* = 0.005) [6]. Therefore, a simple and easy applicable system in predicting mortality for PPU patients may provide reduction in mortality rates.

As a limitation, our study population was only 227 but this number was noticeable when compared with other studies in the literature except cohort study of Moller [5,11,13,15]. Secondly, this was a retrospective analysis, and its prospective confirmation is evitable.

## Conclusion

POMPP is a very simple and appropriate scoring system for clinical practice that may allow surgeon to perform a rapid analysis and may help in predicting mortality rate in PPU with its construction based on objective data.
